# A Case of Ceftaroline-Associated Thrombocytopenia

**DOI:** 10.7759/cureus.47580

**Published:** 2023-10-24

**Authors:** Ashwin Jagadish, Samra Hassan, Shahnawaz Notta, Venkata Vedantam, Neethu Vedantam

**Affiliations:** 1 Internal Medicine, East Tennessee State University James H. Quillen College of Medicine, Johnson, USA; 2 Infectious Diseases, East Tennessee State University James H. Quillen College of Medicine, Johnson, USA

**Keywords:** adverse event, infectious diseases, internal medicine, thrombocytopenia, ceftaroline

## Abstract

Ceftaroline is a fifth-generation cephalosporin that can be used for the treatment of serious infections caused by methicillin-resistant *Staphylococcus aureus* (MRSA). A rare adverse effect of ceftaroline therapy is thrombocytopenia. Our case involves a 45-year-old male with active intravenous drug usage who presented with persistent fever, lower back pain, and left elbow pain. His bloodcultures were found to be positive for MRSA. He was initially started on vancomycin; subsequently, the antibiotic was changed to daptomycin and ceftaroline, as vancomycin failed to clear the bacteremia. Seven days after initiation of ceftaroline, it was unintentionally discontinued by the electronic health record. Following its resumption two days later, the patient started having epistaxis accompanied by an acute drop in his platelet count from 422,000 cells/µL to less than 2,000 cells/µL. The ceftaroline therapy was discontinued, and he received a platelet transfusion. However, daptomycin was continued, resulting in successful resolution of his bacteremia. The patient's platelet count at discharge improved to 582,000 cells/µL. The patient was diagnosed with ceftaroline-induced thrombocytopenia, and it was added to his list of allergies.

## Introduction

Thrombocytopenia is a very common hematologic abnormality seen in patients admitted to hospital [1]. Oftentimes, it is mild-to-moderate and will not result in any clinically significant bleeding [2]. However, when severe, it can cause spontaneous major bleeding, which can be fatal to the patient [2]. Various causes account for thrombocytopenia in hospitalized patients, including drug-induced, immune-mediated, sepsis-related, and disseminated intravascular coagulation (DIC) [1]. Sometimes, multiple causes coexist, resulting in diagnostic difficulty [1]. We present a case of thrombocytopenia associated with ceftaroline, in which the nadir platelet count was lower than the levels that have been reported thus far.

## Case presentation

**Figure 1:**
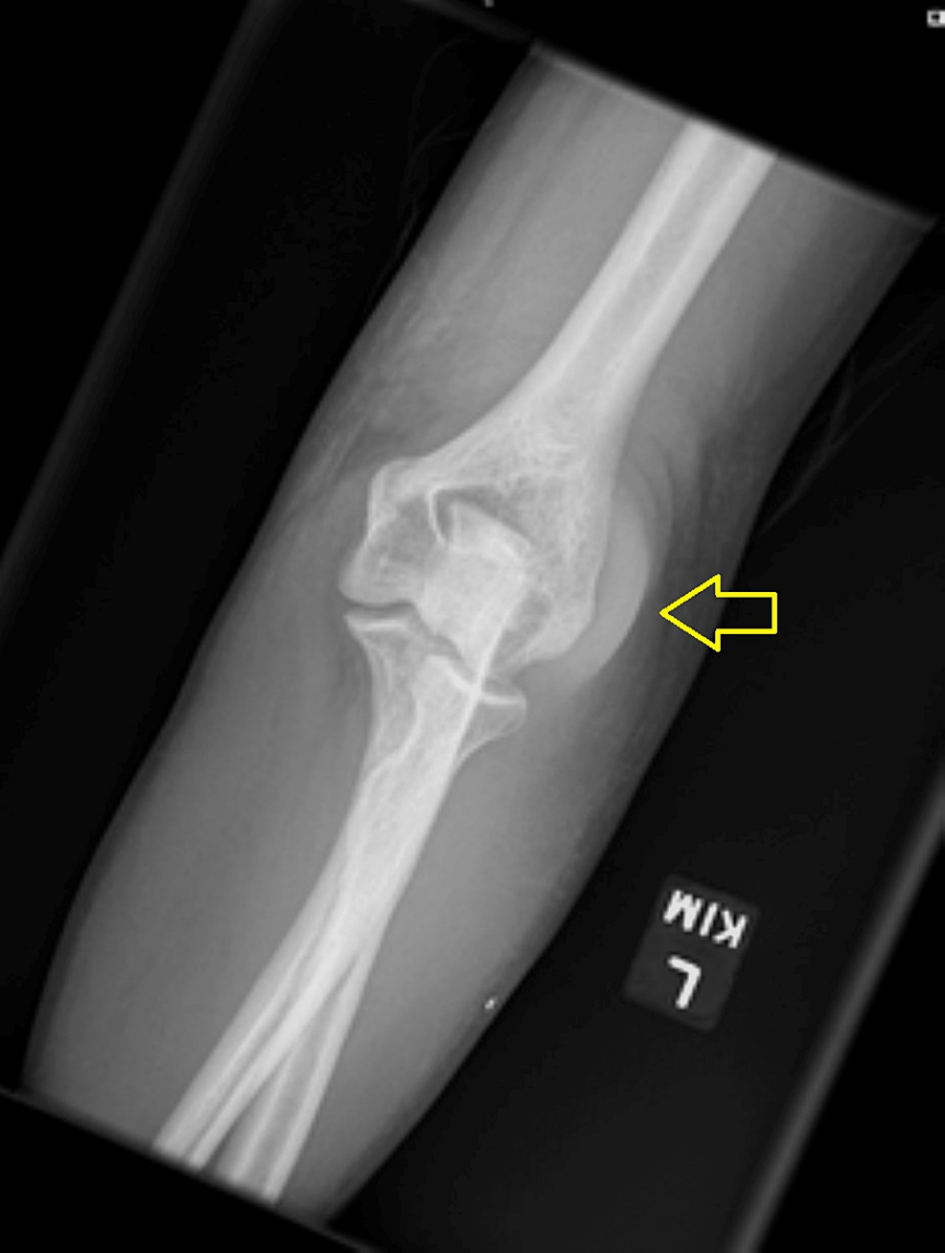
X-ray of left elbow suspicious for olecranon bursitis The arrow indicates the location of olecranon bursitis.

A 45-year-old male patient, with active intravenous drug usage, presented to the emergency department due to persistent low-grade fever, low back pain, and left elbow pain. On admission, he had a temperature of 100.8 °F, white blood cell count of 50,800 cells/µL (normal range: 3,500-11,000 cells/µL), red blood cell count of 4.38 million cells/µL (normal range: 4.22-5.82 million cells/µL), and platelet count of 321,000 cells/µL (normal range: 150,000-400,000 cells/µL). Left elbow x-ray indicated soft tissue swelling suspicious for olecranon bursitis (Figure 1). Magnetic resonance imaging of the cervical, thoracic, and lumbar spine revealed multiple small epidural abscesses which, per neurosurgery, were too small to be operable.

Blood cultures and olecranon bursa aspirates demonstrated methicillin-resistant *Staphylococcus aureus* (MRSA), with sensitivity to vancomycin, ceftaroline, and daptomycin. As a result, intravenous (IV) vancomycin was initiated. Initial echocardiogram did not reveal valvular vegetations. On hospital day four, the patient's bacteremia remained persistent. Therefore, in addition to continuing vancomycin, IV ceftaroline dosed at 600 mg thrice daily was started. On hospital day six, vancomycin therapy was discontinued and replaced by IV daptomycin due to the persistence of bacteremia. Repeat echocardiogram did not reveal valvular vegetations. During this time, he developed reactive thrombocytosis, with a peak platelet count of 952,000 cells/µL. On hospital day 11, ceftaroline therapy was unintentionally discontinued, since the initial order was for 21 doses. At the time of discontinuation, the platelet count had decreased to 422,000 cells/µL.

**Figure 2:**
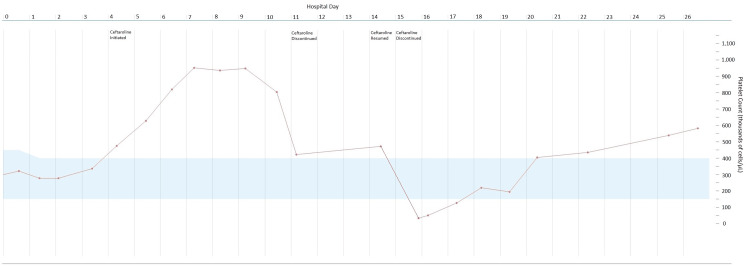
Platelet count during the hospital course

Three days after discontinuation, the platelet count had increased to 477,000 cells/µL. On this day, ceftaroline was restarted; one day after the reintroduction of ceftaroline, he started having epistaxis. Laboratory testing revealed a platelet count of less than 2,000 cells/µL and an immature platelet fraction of 0.0% (normal range: 1.0%-9.3%). The low platelet count was confirmed by peripheral blood smear. Additional peripheral blood smear findings included normocytic, normochromic red blood cells and normal neutrophils, lymphocytes, and monocytes. Additionally, platelet factor 4 antibodies were negative. His coagulation profile was normal, ruling out DIC. Due to the sudden decline in platelet count following readministration, ceftaroline was discontinued, and a platelet transfusion was provided. Only daptomycin monotherapy was continued for the remainder of the hospital course. The patient's platelet count gradually began to increase over the course of the next few days. At discharge, nine days after discontinuing ceftaroline, his platelet count was 582,000 cells/µL, white blood cell count was 9,500 cells/µL, and red blood cell count was 2.84 million cells/µL. The patient was diagnosed with drug-induced thrombocytopenia (DIT) due to ceftaroline, and it was added to his list of allergies. The platelet trend can be seen in Figure 2.

## Discussion

Ceftaroline is a parenterally administered fifth-generation cephalosporin that has broad coverage against both Gram-positive and Gram-negative bacteria [3]. A distinguishing attribute of this cephalosporin is its ability to cover MRSA [3]. Ceftaroline has a similar mechanism of action to other cephalosporins [3]. It has a beta-lactam ring that binds to bacterial penicillin-binding proteins, resulting in the inhibition of cell wall synthesis and subsequent bacterial death [3]. Common side effects of ceftaroline include diarrhea, nausea, constipation, vomiting, and injection-site erythema [4]. A rare adverse event, thrombocytopenia, was seen in fewer than 2% of individuals during phase III clinical trials [5].

DIT is a commonly encountered adverse effect in hospital settings [6]. Often, the offending medication is difficult to identify, so it is important for clinicians to be aware of medications that can produce this effect. Several mechanisms of DIT have been elucidated, including non-immune, immune, direct bone marrow suppression, or the formation of antibodies, resulting in increased consumption of platelets, as seen in heparin-induced thrombocytopenia [6]. Hapten-mediated drug-dependent antibodies are presumed to be responsible for thrombocytopenia associated with penicillins and certain cephalosporins [6]. Immune thrombocytopenia typically occurs five to 10 days after initiation of the drug [6], and it can be quite dramatic and severe upon rechallenge, as in our case.

The onset of thrombocytopenia five to 10 days from the start of therapy and the dramatic drop upon reintroduction, along with prompt resolution upon discontinuing the suspected medication, confirmed ceftaroline to be the perpetrator in our case. The Naranjo scale for adverse drug events revealed a "definite" rating based on a score of 10 points. Points were granted for previous conclusive reports of this adverse drug event (+1), adverse event appearing after drug administration (+2), adverse reaction improving after drug cessation (+1), adverse reaction appearing after drug readministration (+2), lack of alternative possibilities for the reaction (+2), lack of reappearance of the reaction with placebo (+1), and confirmation of the reaction by objective evidence (+1). We believe that the initial decline in platelet count is attributed to ceftaroline, especially since the count increased following the initial discontinuation. Upon resuming ceftaroline, there was a dramatic decline in the platelet count to less than 2,000 cells/µL. In this case, daptomycin can be considered a "placebo" for consideration of the Naranjo scale, since antibiotic therapy was required to treat the patient's underlying bacteremia. There were also no other possibilities for this reaction. While daptomycin could cause thrombocytopenia, the patient's platelet counts improved while receiving monotherapy. Throughout the hospitalization, the patient's home medication - buprenorphine - was continued. While hospitalized, he was started on docusate, polyethylene glycol, gabapentin, lisinopril, and metoprolol. The platelet count remained stable with these medications. Additionally, testing for platelet factor 4 antibodies and coagulation panel were negative ruling out heparin-induced thrombocytopenia and DIC. Finally, the peripheral blood smear provided objective evidence of thrombocytopenia.

Although hematologic abnormalities, including neutropenia, have been reported with ceftaroline, to our knowledge, this is only the third reported case of thrombocytopenia [5,7], apart from a retrospective review of 74 patients, which showed five cases of thrombocytopenia with a median onset at seven days and median nadir of 67,000 cells/μL [8]. Another study of 12 patients described a 58% discontinuation rate from hematological toxicity but only one case of thrombocytopenia [9]. To our knowledge, this is only the second case to establish a definite association between ceftaroline therapy and thrombocytopenia.

## Conclusions

Although thrombocytopenia is a commonly encountered hematologic abnormality in the hospital, it is very important to know the exact etiology, as the reversal of platelet count is dependent on withdrawing the offending stimulus. Ceftaroline can be very rarely associated with severe thrombocytopenia, and it is very important for clinicians to keep this in their differential diagnosis of thrombocytopenia, as failure to recognize this can be fatal to the patient.
